# Comparison of the Preemptive/Preventive Effect of Dexmedetomidine and Ketorolac on Post-operative Pain of Appendectomy Patients: A Randomized Clinical Trial

**DOI:** 10.5812/aapm-146868

**Published:** 2024-12-16

**Authors:** Sepideh Pakniyat, Ghasem Mousavi, Hashem Jarineshin, Fereydoon Fekrat, Narjes Sabet, Alireza Abdullahzadeh-Baghaei

**Affiliations:** 1 Anesthesiology, Critical Care and Pain Management Research Center, Hormozgan University of Medical Sciences, Bandar Abbas, Iran

**Keywords:** Dexmedetomidine Hydrochloride, Ketorolac, Analgesia (Patient-Controlled), Pain (Postoperative)

## Abstract

**Objectives:**

The primary objective was to test the hypothesis that the preemptive/preventive effect of Dexmedetomidine would attenuate the post-operative pain more effectively compared to ketorolac and control groups.

**Methods:**

This study was conducted in Shahid Mohamadi Hospital. Sixty patients undergoing appendectomy operations were randomized in 3 groups. Group A received intravenous Dexmedetomidine bolus (1 μg/kg) and infusion (0.5 μg/kg/h). Group B received slow intravenous bolus ketorolac 30 mg. Group C was the control group. Post-operatively fentanyl (5µg/mL) as patient control analgesia (PCA) was provided only on demand. The primary outcome was the Visual Analogue Scale (VAS) pain scores recorded at 1, 3, 6, 12 and 24 hours postoperatively. The secondary outcome was the 24-hour cumulative fentanyl PCA dose. Tertiary outcomes; changes in blood pressure, heart rate, body temperature, SpO_2 _perioperatively. Quaternary outcomes were PONV, shivering.

**Results:**

In the Dexmedetomidine group the mean ± SD pain VAS scores 1.15 ± 1.98 and 0.95 ± 1.76 were significantly lower at 12 and 24 hours after operation (P = 0.004 and P = 0.003) compared to the other two (ketorolac and control) groups. The cumulative volume dose of fentanyl PCA 21.35 ± 11.77 mL was less in the Dexmedetomidine group compared to ketorolac (28.35 ± 9.82 mL, P = 0.629) and control (40.35 ± 12.90 mL, P = 0.003) groups.

**Conclusions:**

Preemptive/preventive effects of Dexmedetomidine were greatest after operation compared to the ketorolac and control groups in the terms of pain scores and amount of analgesia needed postoperatively.

## 1. Background

Pain is recognized as a common side effect of surgery. Postoperative pain is a complex physiological reaction to tissue damage, which may result in persistent chronic pathological changes in the body due to untreated acute postoperative pain (1). Preemptive and preventive approaches have been proposed in order to prevent a central sensitization by blocking the pre-, intra- or post-operative nociceptive inputs from injured tissue (2). The use of different modalities of pain control have been stressed on in order to decrease the use of opioids post operatively, because of their troubling side effects that have accompanied morbidity and mortality in long term outcomes (3). The short term outcomes of preventive analgesia include a faster recovery period with less post traumatic pain, while the long term outcomes include attenuated chronic pain, more patient satisfaction, improved recovery quality and time (4). Different agents have been used for preemptive/preventive analgesia in appendectomy such as intravenous ketamine (5), lidocaine (6). In a recent review article preventive analgesia for appendectomy operations was studied none of the applied methods were recommended except paracetamol and NSAIDS (3), but systemic dexmedetomidine in these type of operations has not been studied yet.

## 2. Objectives

Therefore, this research aimed at comparing the preemptive and/or preventive impacts of dexmedetomidine with ketorolac on pain after appendectomy.

Dexmedetomidine is an imidazole derivative of dimethyl-phenyl-ethyl. At physiologic pH (7.2 to7.4) two types of imidazole, including (1) neutral, and (2) protonated is found (7). Overall, useful implications of analgesic action of dexmedetomidine in multimodal analgesia have been demonstrated (8).

Ketorolac a known non-steroidal anti-inflammatory drug (NSAID) with strong analgesic effects, derived from heteroaryl acetic acids which is mostly used for moderate-to-severe postoperative pain treatment (9). Ketorolac preemptive analgesic effects have been shown in maxillar surgeries (10).

## 3. Methods

The present prospective, parallel, double-blind, placebo-controlled cohort research was confirmed (protocol number IR.HUMS.REC.1397.047) by the Ethics Committee of Hormozgan University of Medical Sciences and Shahid Mohammadi Hospital, Iran (Chairperson Prof. Dr. Hossein Farshidi) on 5 March 2018. It was also registered at the Iranian Registry of Clinical Trials IRCT20140615018091N9.

For calculating the sample size for each group one-sided analysis of variance was used considering mean values of the VAS scores one hour after operation from a prior pilot study which were as following µ_1_ = 2.8, µ_2_ = 5, µ_3_ = 7 for the dexmedetomidine, ketorolac and control groups respectively. Sample size calculations was applied by taking the α = 0.01, β = 0.1, σ = $\sqrt{MSE\;}$= 2.55. We calculated a λ from the above indices and obtained a non-central distribution χ^2^ equal to 12.66 and the Δ the number of cases for each group was 15. For a possibility of drop outs we choose a number of 20 patients for each group (NCSS software).


$$∆\;=\;\frac{1}{\sigma^{2}}\sum_{i=1}^{k}{{(\mu }_{j}-\;\overset{-}{\mu })}^{2}$$

$$\overset{-}{\mu }=\frac{1}{k}\sum_{i=1}^{k}\mu_{j}\;$$

$$X_{k-1}^{2}\left(X_{\alpha ,\;k-1}^{2}\left|\lambda \right.\right)=\;\beta$$

**Figure 1.**
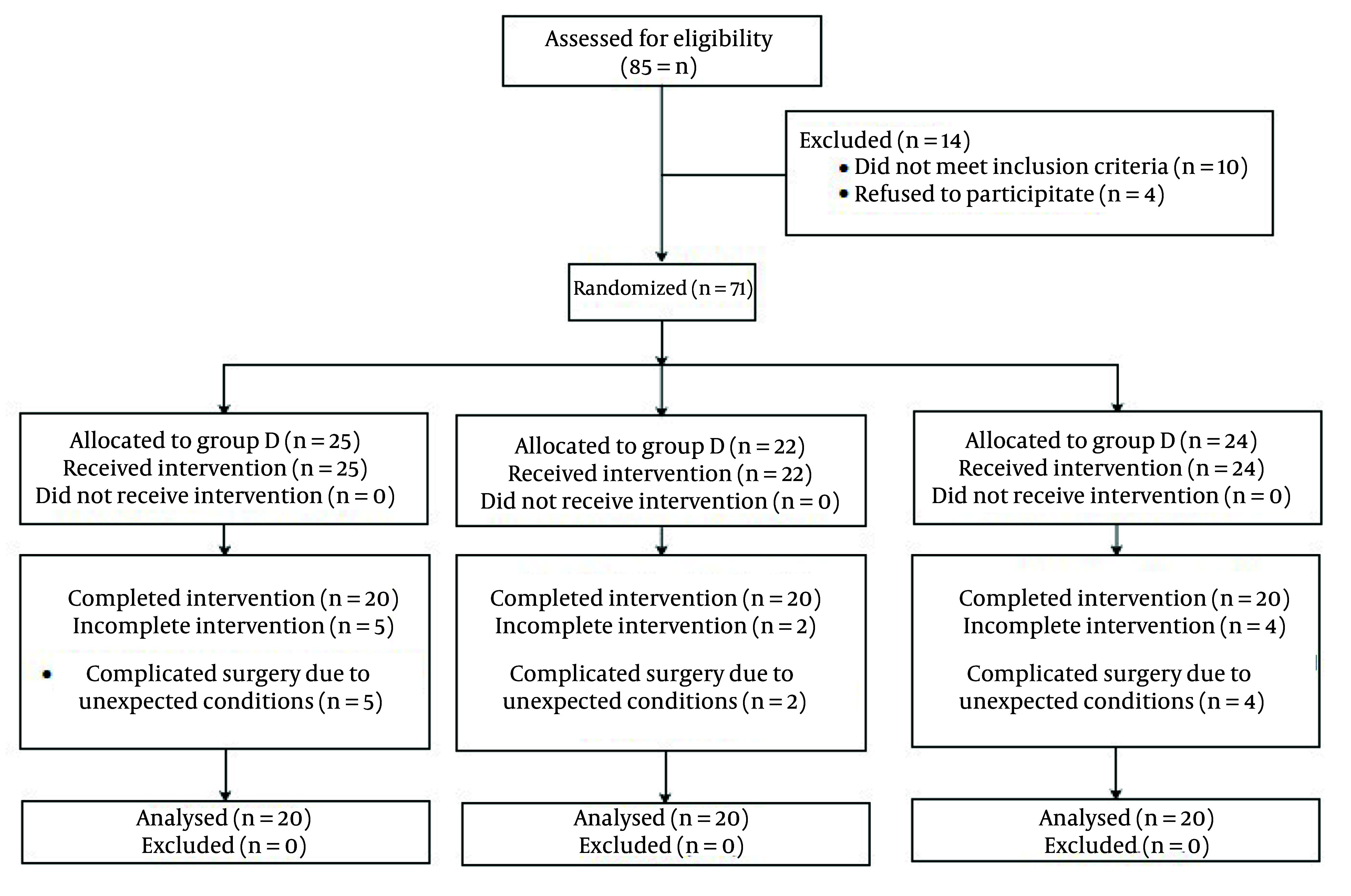
Consort flow diagram

This study was conducted on 60 patients undergoing conventional open appendectomy operation. All the patients were diagnosed as with non-perforated appendicitis. The inclusion criteria included the age range of 18 to 65-years; American Society of Anesthesiology (ASA) class I and II. The subjects were divided in three groups including dexmedetomidine (group D), ketorolac (group K), and control (group C) groups, based on a computer-designed block randomization chart (Consort flow diagram in Figure 1). Randomization was applied on individual units on a block table with 1:1 allocation and a block size 60. This was considered because we calculated a sample size of 20 for each group. For each recruited candidate a sequence number coded sealed envelope containing a sheet paper with an A (group D), B (group K) or C (group C) letter indicating either of three protocol agents. Allocation concealment was carried out by the primary anesthesiologist in this study.

On the other hand, the exclusion criteria were (1) history of allergy to the prescribed medications; (2) addiction or elicited use of drugs and analgesics; (3) complicated appendicitis; (4) neurological disorders; (5) neuropathic pain; (6) treatment with analgesic or anti-inflammatory medications; (7) pregnancy or breastfeeding; (8) unwillingness to participate in the study. Fourteen patients were excluded from the 85 due to the above criteria. From the 71 patients assigned to the study groups 11 were omitted due to complicated surgery and unexpected conditions during the operation (Figure 1). 

Standard monitoring was performed using an anesthesia monitoring system (Spacelabs 90387, USA), including pulse oximetry, non-invasive blood pressure (NIBP), and electrocardiography (ECG) monitoring for all patients. The patients’ basic vital signs were recorded after resting for five minutes. After recording the basic vital signs, an intravenous infusion of Ringer’s solution (5 mL/kg) was used before administering the agents.

The study and control groups drugs were prepared and coded by the anesthesia technician at the drug preparation room. For each patient who was randomly assigned to one of the 3 groups a package of 50 mL syringes A_1&2 _or B_1&2 _or C_1&2 _was delivered accordingly to the anesthesiology resident who was informed to apply the syringes 1 and 2 sequential manner according to the protocol of the study.

The prepared drugs were as follows; dexmedetomidine hydrochloride for group D [syringes A_1&2_] (Precedex, Hospira Inc., Lake Forest, IL 60045, USA); ketorolac for group K [syringes B_1_] (Alborzdaru, Tehran, Iran); and normal saline 0.9% (Samen pharmaceuticals, Mashhad, Iran) or placebo for group K [syringes B_2_] and group C [syringes C_1&2_]. The syringes (50 mL) were labeled as A_1&2_, B_1&2 _and C_1&2_; respectively for blinding the data collector anesthesiology technician). They were infused be a syringe pump (model 8713030, B Braun, Germany).

Syringe A_1_ contained 1 μg/kg of dexmedetomidine diluted with normal saline 0.9% to 50 mL. Syringe B_1_ contained 30 mg of ketorolac diluted with normal saline 0.9% to 50 mL. Syringes B_2_ and C_1& 2 _contained 50 mL of normal saline 0.9%.

After the first set of syringes was injected within 10 minutes before anesthesia induction, induction of general anesthesia was done and the second set of syringes was infused at a rate of 0.5 mL/kg/h until skin closure. The second set of syringes was coded as follows; syringe A_2_ (dexmedetomidine 1 µg/mL), syringe B_2_ and C_2_ (normal saline 0.9%).

Anesthesia induction was initiated with premedication of 0.05 mg/kg midazolam, and 2 µg/kg of fentanyl, and was continued with 1 mg/kg of lidocaine, 2 mg/kg of propofol, atracurium 0.6 mg/kg intravenously and the patients were intubated 3 minutes afterwards by the anesthesiology resident.

Anesthesia was maintained by infusion of 100 mcg/kg/min of propofol with a fresh gas flow of 6 L/min with N_2_O and O_2_ (50:50); end-tidal CO_2_ (ETCO_2_) was also maintained at a 30 - 35 mmHg. Intraoperative fluid dosing was applied to calculate the fluid requirements during surgery. Accordingly, a Ringer’s solution was used. Neostigmine (0.05 mg/kg) was used to antagonize muscle relaxation and 0.02 mg/kg atropine sulphate.

The patients’ vital signs (blood pressure, heart rate, arterial blood oxygen saturation, body temperature) were noted in a questioner after five minutes of resting on the operating table (11) and immediately after endotracheal intubation, the vital signs recordings were continued at sequential time points as follow; 5 - 10 - 15 - 20 - 30 - 40 after intubation, and every 10 min to the end of operation. During the operation the rooms temperature was kept in the range of 22 - 23 degrees Celsius (12). In patients with shivering after the operation, the intensity of shivering was recorded and managed by warming; if needed, 25 mg of meperidine (Pethidine 50 mg/mL, Exir Pharmaceuticals Co., Iran; www.exir.co.ir) was applied intravenously. After the operation the patient control analgesia (PCA) pump (Automed 3400, ACE MEDICAL^©^, Bomun-ro, Seongbuk-gu, Seoul, South Korea, www.ace-medical.com) was applied. The PCA medication was applied via a 100 mL 0.9% normal saline solution, containing 500 µg fentanyl. The bolus demand dose was set at 10 mcg of fentanyl with a lockout time of 10 minutes. The PCA pump was used in all groups for 24 hours. Pain intensity related Visual Analogue Scale (VAS) scores were recorded post operatively at the 1, 3, 6, 12 and 24 h after the operation. For cases with a VAS pain score above three, 100-mg diclofenac suppository (Iran Najo Pharmaceutical Co., Tehran, Iran) was administered to treat the breakthrough pain. The cumulative dose of fentanyl PCA was recorded. The patients and the anesthesiology resident in charge of recording the information were blinded to the type of applied medications.

The data were analyzed in SPSS version 19. Descriptive tests were used (mean, standard deviation, percentiles). For sample distribution Kolmogorov-Smirnov test was used. Chi-square test for differences in nominal demographic data. For the difference among three groups, normally distributed data with one-way ANOVA (post hoc Tukey's test) and non-normal distributed data with Kruskal Wallis test (post hoc Dunn's test). Friedman test for repeated measurements over time within each group. P-values of smaller than 0.05 were regarded significant.

## 4. Results

The dexmedetomidine group included 12 (60%) male and 8 (40%) female patients, while the ketorolac and control groups each consisted of 15 (75%) male and 5 (25%) female patients. However, other demographic characteristics (mean age, height, weight, and ASA class) showed no significant differences among the three groups (Table 1). 

**Table 1.** Demographic Information of Dexmedetomidine, Ketorolac, and Control Groups ^a^

Variables	Groups			Statistical Test		P-Value
	D	K	C	Chi-Square	ANOVA	
**Age (y)**	30.35 ± 9.97	25.65 ± 6.23	26.15 ± 11.69	5.276		0.071
**Height (cm)**	165.75 ± 9.74	167.40 ± 12.43	169.90 ± 8.35	1.316		0.518
**Weight (kg)**	66.20 ± 11.24	69.65 ± 16.40	65.65 ± 14.12		0.474	0.625
**Sex**				1.429		0.490
Male	12 (60.0)	15 (75.0)	15 (75.0)			
Female	8 (40.0)	5 (25.0)	5 (25.0)			
**ASA**				3.111		0.310
I	17 (85.0)	19 (95.0)	20 (100.0)			
II	3 (15.0)	1 (5.0)	0 (0.0)			

^a^ Values are presented as mean ± SD or No. (%).

**Figure 2.**
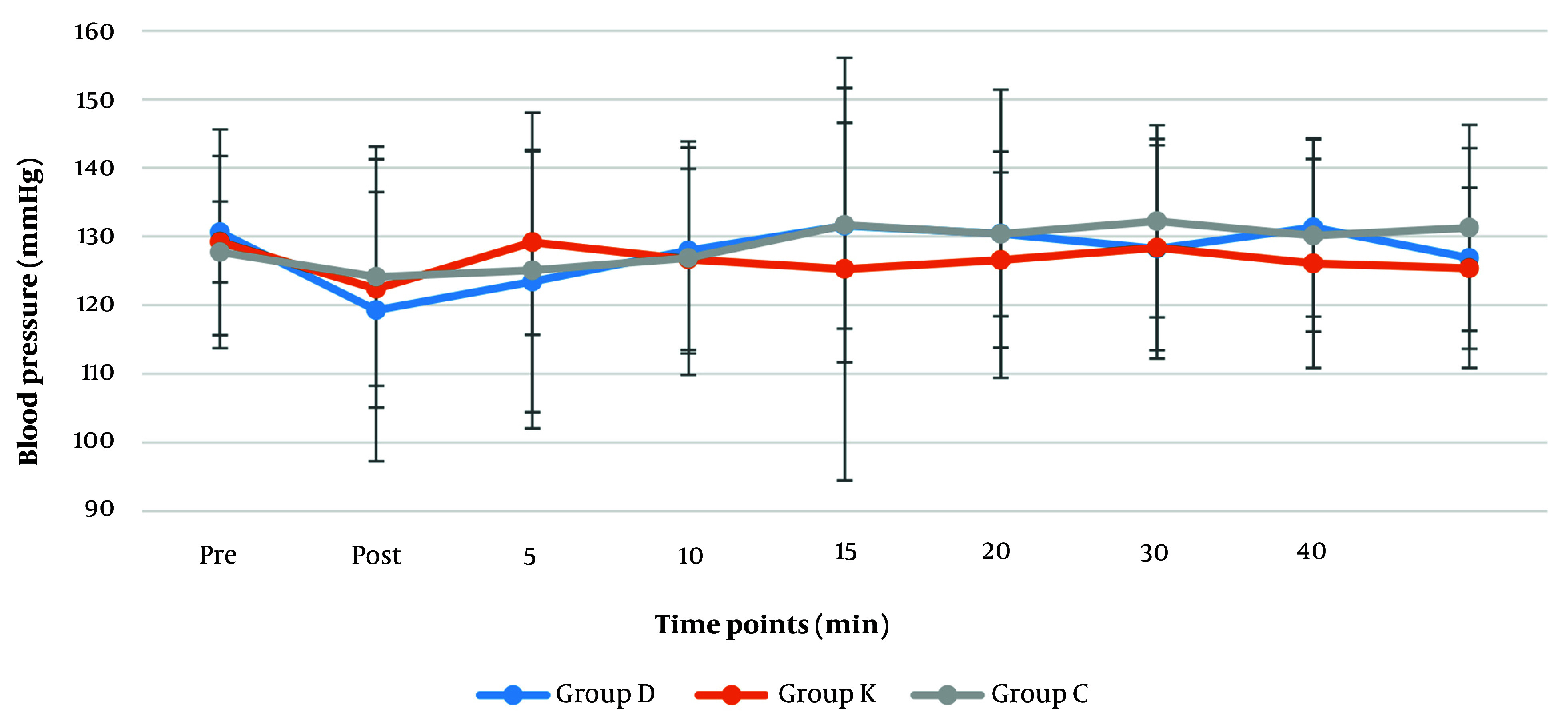
Perioperative distribution of systolic blood pressures (mmHg) perioperatively (pre: Pre-induction, post: Post-intubation, 5 to 40 minutes' post-intubation and recovery time). Values are expressed as mean ± SD.

**Figure 3.**
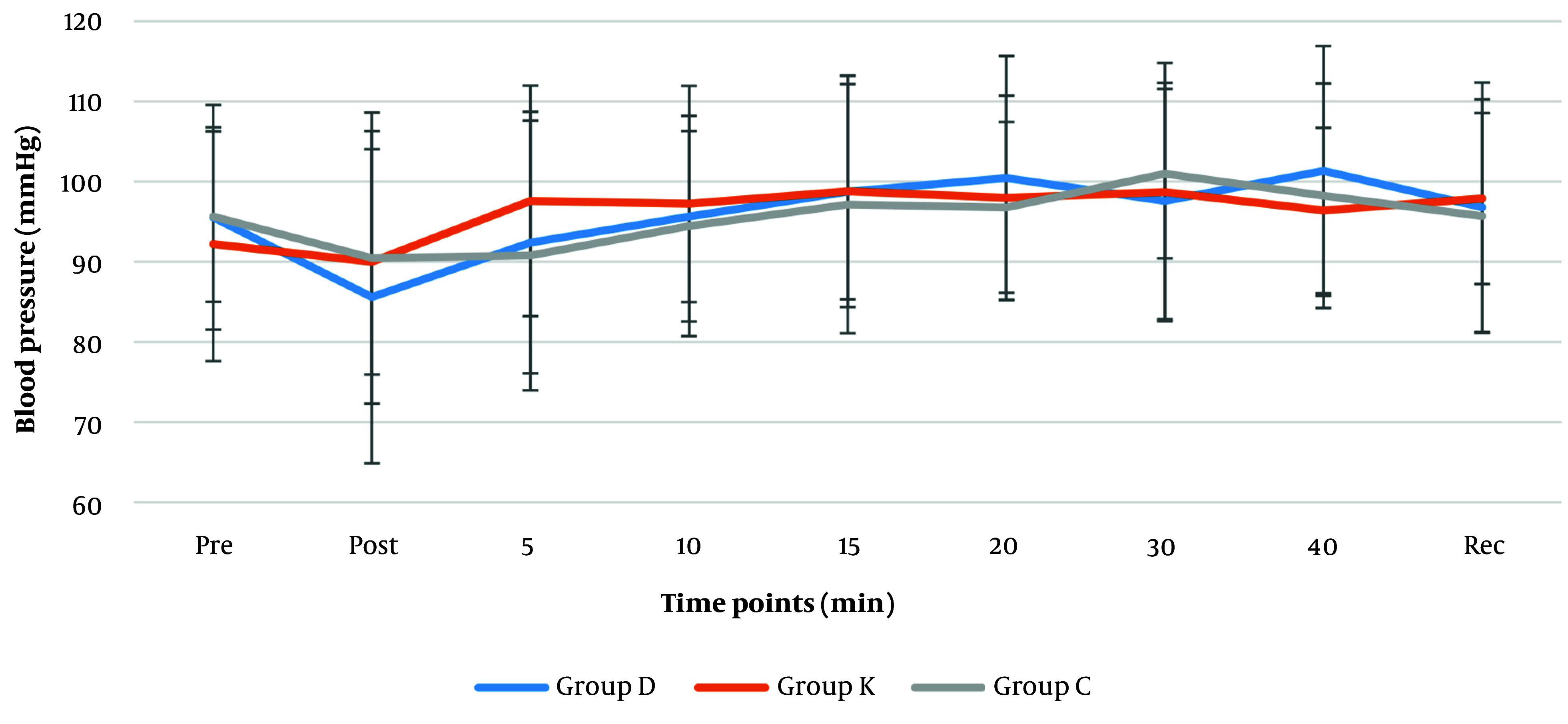
Distribution of mean arterial blood pressures (mmHg) perioperatively (pre: Pre-intubation, post: Post-intubation, 5 to 40 minutes post-intubation and recovery time). Values are expressed as mean ± SD.

Comparing the systolic (120 - 130 mmHg), diastolic (84 - 86 mmHg) and mean arterial blood pressures (MAP) (85 - 100 mmHg) (Figures 2 and 3) revealed no significant differences among the groups. According to Friedman test for repeated measurements over time, a significant change was found in MAP reading of the dexmedetomidine group (P = 0.033). Reduction of MAP to (85.6 mmHg) was reported after intubation in dexmedetomidine group, which gradually increased up to (101.4 mmHg) 20 minutes later; the remaining MAP measurements in this group were only slightly higher than the pre-intubation MAP (Figures 3). 

**Figure 4.**
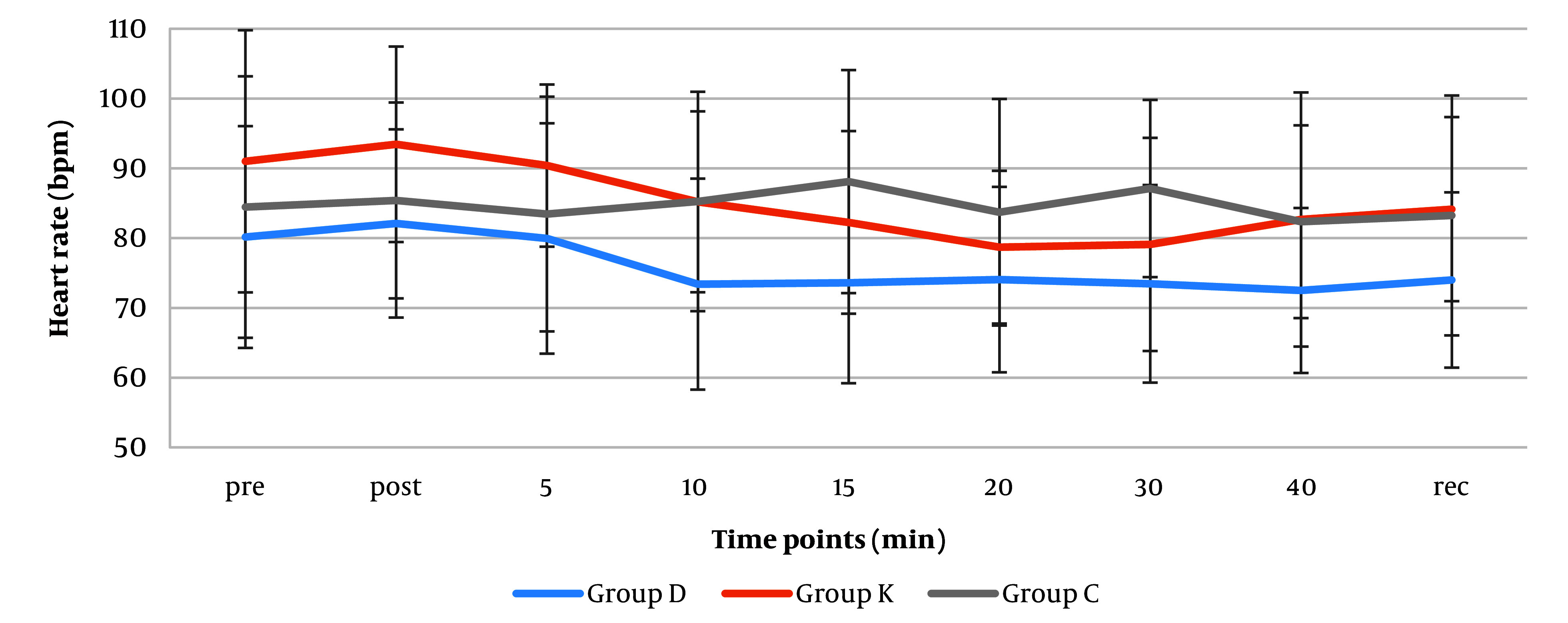
Distribution of the mean heart rate (beats/minute) perioperatively (pre: Pre- intubation; post: Post-intubation, 5 - 40 minutes post- intubation, and during recovery). Values are expressed as mean ± SD.

The mean heart rate changes pre-intubation, post-intubation, 5, 20, and 40 minutes after intubation, and during recovery showed no significant differences in the groups (Figure 4). However, mean heart rates were significantly lower in the dexmedetomidine group than the ketorolac group at post-intubation (82 vs 93 beats/minute), 10th minutes (73 vs 85 beats/minute) (P-values: 0.033 and 0.024 respectively). The mean heart rates were also significantly lower in the dexmedetomidine group than the control group at 10 (73 vs 85 mean HR), 15 (74 vs 88 mean HR), and 30 (73 vs 85 mean HR) minutes after induction (P = 0.022, 0.010 and 0.015 respectively). Overall the mean heart rates in this group (74 mean HR) were clinically less than the other ketorolac and control groups (84 and 83 mean HR). According to Friedman test, there was a significant change over time in both Dexmedetomidine (74 - 80 mean HR change range) and ketorolac (79 - 90 mean HR change range) groups (P = 0.002 and P < 0.001, respectively).

**Figure 5.**
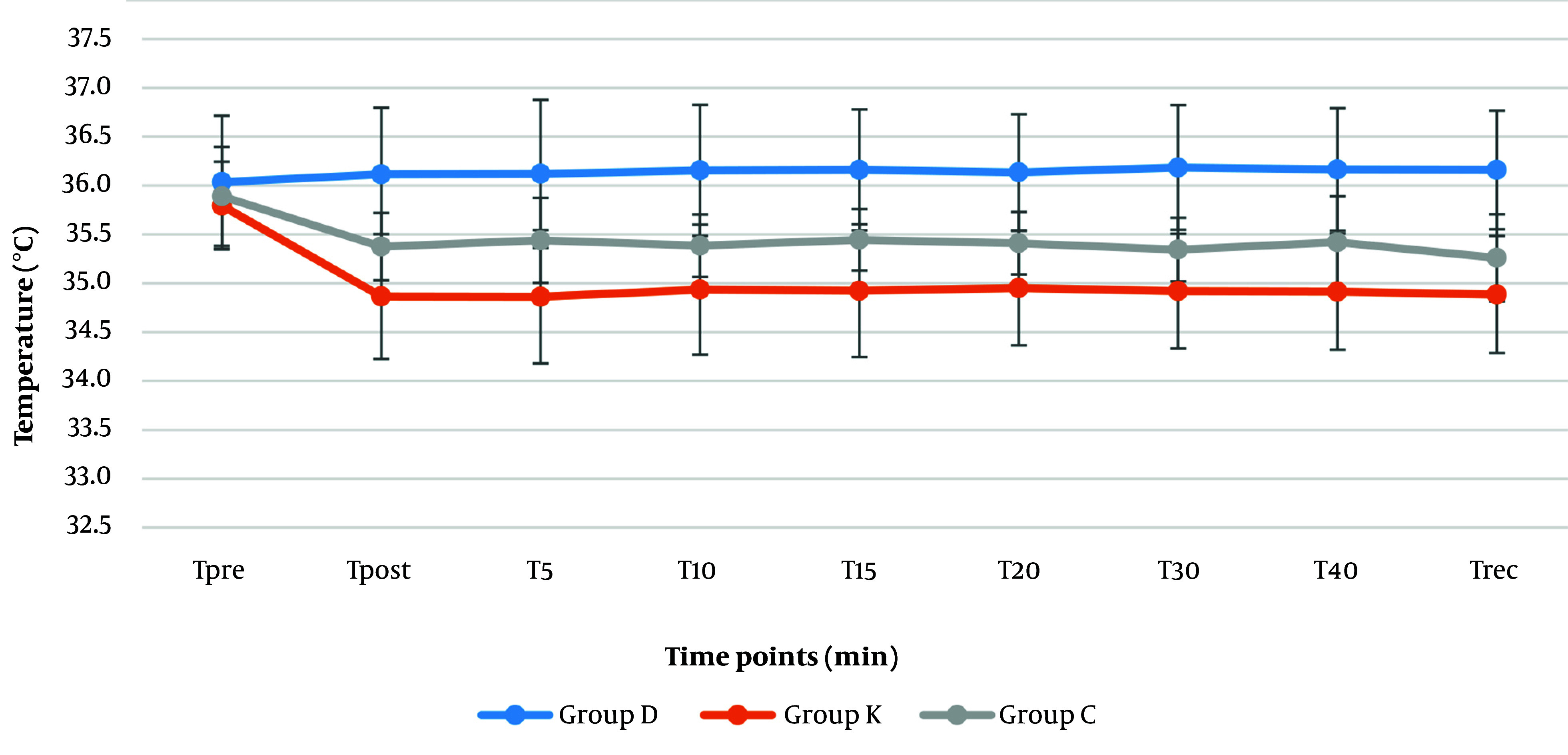
Distribution of mean temperature (°C) perioperatively (pre: Pre-intubation, post: Post-intubation, 5 - 40 minutes post-intubation and during recovery) in the dexmedetomidine (group D), ketorolac (group K) and control (group C). Values are expressed as mean ± SD.

Higher levels of temperature were recorded in the dexmedetomidine group (36 - 36.2℃) than the ketorolac (35.9 - 34.9℃) and control groups (35.3 - 35.9℃) perioperatively (P < 0.001) (Figure 5). Unlike the dexmedetomidine group the temperature showed a significant declining trend towards the end of surgery in the ketorolac group ketorolac (34.9℃) (P = 0.037) and control (35.3℃) (P = 0.001) groups.

Furthermore, the results showed that the mean partial oxygen saturation (SpO_2_) was 99 - 100% in all groups perioperatively. No significant difference was observed among the groups. Also, there was no significant change in SpO_2_ at different intervals in either of the groups.

**Figure 6.**
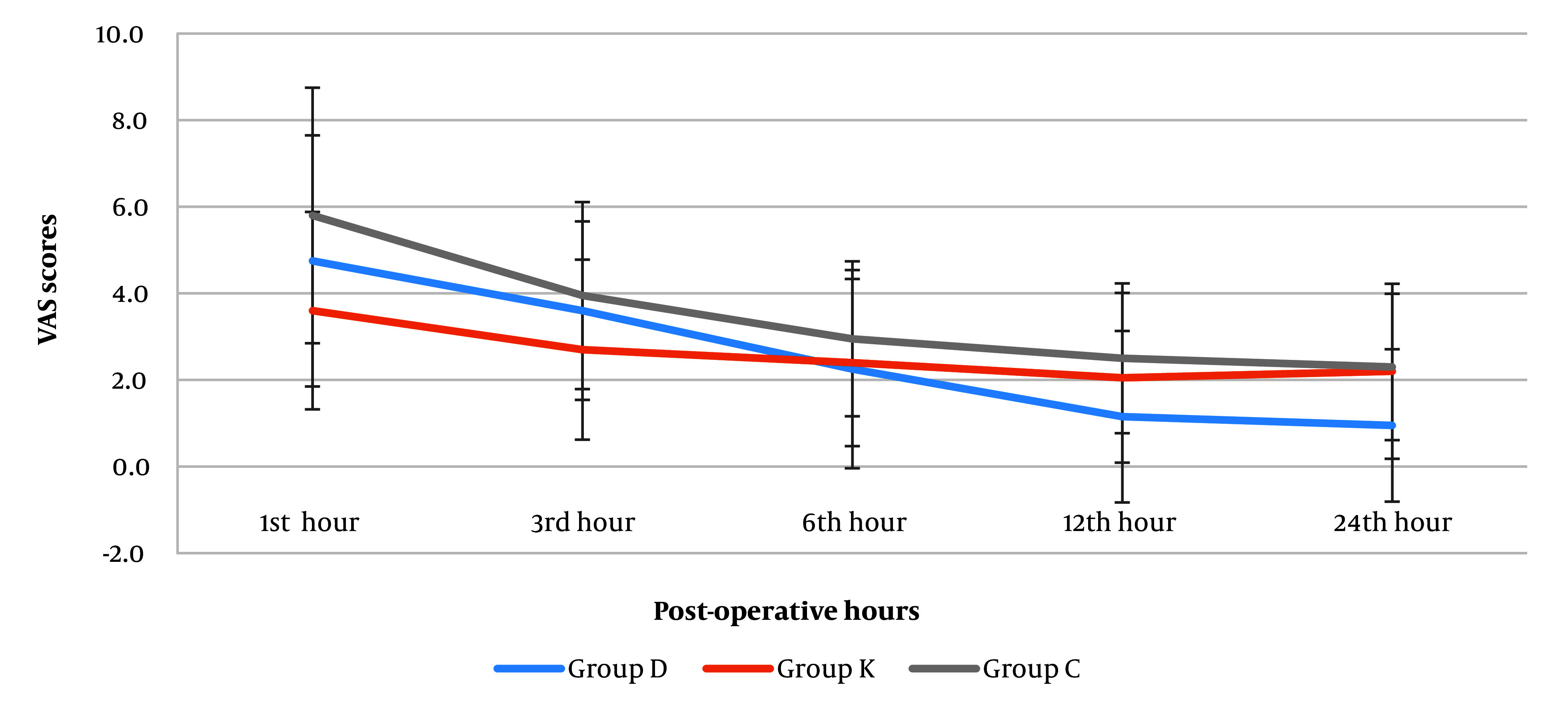
Postoperative distribution of pain intensity based on Visual Analogue Scale (VAS) (1, 3, 6, 12, and 24 hours postoperatively) in the dexmedetomidine (group D), ketorolac (group K), and control (group C) groups. Data are presented as mean ± SD.

The VAS pain scores were higher in the early hours after the operation and decreased gradually. The mean VAS pain scores were not significantly different among the groups at 1, 3, and 6 h after operation (Table 2 and Figure 6). In the first and third hours after operation the mean VAS pain scores were lower in the ketorolac group compared to control as well as dexmedetomidine groups, which was not statistically significant. However, the pain intensity was significantly lower in the dexmedetomidine group compared to the ketorolac and control groups at 12 hours (D: 1.15 vs. K: 2.05, C: 2.50) and 24 hours (D: 0.95 vs. K: 2.20, C: 2.30) postoperatively (P = 0.004 and P = 0.003). Additionally, no significant difference was found between the ketorolac and control groups at these intervals. Friedman test indicated a significant reduction in mean VAS pain intensity towards the 24 hours postoperatively in all groups (D: 4.75 to 0.95, K: 3.60 to 2.20, C: 5.80 to 2.30) (P < 0.001).

**Table 2.** Postoperative Pain Intensity Based on Visual Analogue Scale in the Dexmedetomidine (Group D), Ketorolac (Group K), and Control (Group C) Groups

Variables	Pain Intensity VAS				Post Hoc Between Groups (P-Values)		
	D	K	C	P-Value	D-K	D-C	K-C
**Hours after end of operation**							
1	4.75 ± 2.90	3.60 ± 2.28	5.80 ± 2.95	0.053	-	-	-
3	3.60 ± 2.06	2.70 ± 2.08	3.95 ± 2.16	0.084	-	-	-
6	2.25 ± 2.29	2.40 ± 1.93	2.95 ± 1.79	0.258	-	-	-
12	1.15 ± 1.98	2.05 ± 1.96	2.50 ± 1.73	0.004 ^a^	0.014 ^a^	0.001 ^a^	0.195
24	0.95 ± 1.76	2.20 ± 2.02	2.30 ± 1.69	0.003 ^a^	0.008 ^a^	0.001 ^a^	0.552
**PCA use of fentanyl (mL)**	21.35 ± 11.77	28.35 ± 9.82	40.35 ± 12.90	0.002 ^a^	0.629	0.003 ^a^	0.081

Abbreviations: D, dexmedetomidine; K, ketorolac; C, control; PCA, patient control analgesia; VAS, Visual Analogue Scale.

^a^ Significant values.

The frequency and amount of PCA analgesic use revealed that three patients from the dexmedetomidine group did not require fentanyl, although it was not statistically significant (P = 0.100). There was a significant difference between the groups for the average required amount of fentanyl solution (mL) (P = 0.002), which was lowest in dexmedetomidine group (21.35 mL) and the highest in the control group (40.35 mL). The average required concentration of fentanyl was only significantly lower in the dexmedetomidine group in comparison with the control group (P = 0.003; Table 2). 

The need for extra analgesia (diclofenac suppository) for breakthrough pain while receiving fentanyl PCA was reported in two cases from the ketorolac group as well as five patients from the control group, while none in the dexmedetomidine group required extra analgesics; however, the difference was not significantly different (P = 0.058).

Based on our findings, 3 in D group 2 in K group and none in C group had postoperative nausea and vomiting (PONV). The difference between these groups in this regard was not significant (P = 1.000).

Shivering intensity I was reported in two patients from the control group, intensity II was found in one subject in the ketorolac group and a case in the control group, and intensity III was recorded in one patient from the control group. Control patients with shivering intensities II and III required treatment with meperidine (25 mg, i.v.), while only warming was adequate for the rest of the patients who had shivering (P = 0.115).

## 5. Discussion

According to Katz et al. a preventive (preventive analgesia is broader definition of preemptive analgesia) analgesic can be related to an agent when the time duration of this impact is longer than the agents target effect, which has been defined by them as 5.5 times the half-life of that particular agent (2). This means that the process of preventive analgesia should not be a direct result of the agent's analgesic property. Overall in this study the pain intensity was significantly lower in the ketorolac group in the earlier hours after operation but was changed in the favor of the dexmedetomidine group in the later hours after surgery. This may indicate that the dexmedetomidine preventive effect at this time point is almost beyond the 5.5 times its half-life (dexmedetomidine half-life; 2 - 3 hours) (4). However, for ketorolac this did not happen. Because, 22 - 33 hours after the administering ketorolac (ketorolac's half-life 4 - 6 hours which is longer than dexmedetomidine) (13), the VAS scores in the ketorolac group did not attenuate statistically compared to group D and C. Additionally, for maxillofacial surgeries, the mean analgesic duration for ketorolac’s preemptive effect has been defined as 8.9 hours (14) and in two other studies for pain management it has been reported about 6 to 12 hours (15, 16), and this may be an explanation for the low VAS scores in ketorolac group only until the 12 hour postoperatively (Table 2). This may imply the lesser preventive nature of keterolac compared to dexmedetomidine. The extenuative effect of dexmedetomidine in our study 12 and 24 hours postoperatively is in contrast to the findings of previous studies, where dexmedetomidine was shown to be most effective only until the 48th hours postoperatively (17).

The most influential factors for postoperative pain are efficacy and duration of analgesia in the perioperative period, while timing of administration is less effective (18). In our comparative study of dexmedetomidine and ketorolac, efficacy was the primary objective. Considering the different nature of dexmedetomidine and ketorolac in our trial, duration of analgesia exposure was not similar. Dexmedetomidine generally has a shorter half-life than ketorolac; this drug was infused throughout surgeries, providing approximately one-hour exposure in our study.

Although we did not record the duration of surgeries, almost all procedures were performed by the same surgical and anesthesia resident group within 40 - 60 minutes. The pharmacokinetics data of dexmedetomidine are variable concerning its activity onset in different studies. In this regard, a study from Rautela et al. showed that a 10 minutes' interval was required for the onset of dexmedetomidine activity after intravenous administration of bolus dose of 0.5 µg/kg (19). In contrast, AL-Mustafa et al. and Alshawadfy et al. found that an intravenous maintenance infusion of dexmedetomidine (1 µg/kg) over 10 minutes followed by an infusion is effective with lesser side effects compared to a 30 minutes interval dosing (20, 21)

Recently, Simmons and Kuo found that intravenous dexmedetomidine infusion exerts its effects within 15 minutes, while it reaches its peak level after one hour (22). Comparatively the onset of action for intravenous ketorolac in 10 minutes after administration, and peak analgesia is reached at within 75 to 150 minutes (23). Although the onset of action is similar for both agents, their peak effect intervals are different. The pharmacologic effectiveness of ketorolac may extend beyond the time of surgery, whereas the pharmacologic effects of dexmedetomidine are limited to the surgery period. We can assume from this perspective of our results that dexmedetomidine has a greater preventive effect compared to ketorolac, while ketorolac effect is a more preemptive type. Moreover, it has been proposed that NSAIDs, such as ketorolac, should be administered long before the operation; this is because of the peripheral target points of NSAIDs (24, 25). Considering the nature of appendectomy surgery in emergency settings, scheduling is not feasible for ketorolac administration before admitting the patient to the operating room. Moreover, emergency patients are dehydrated, and effective vascular volume is less reliable (26), as hemodynamic changes need to be controlled. We can conclude that our study protocol is adequate for assessing the preemptive and preventive effects of drugs. Furthermore, to determine the appropriate time of drug administration for optimal antinociceptive effects, the peak analgesic effect and preemptive conditioning must be considered (27). Dexmedetomidine has antinociceptive effects on visceral and somatic pain (28) where both type of pains are common findings in appendicitis events.

In our study no difference was seen in the VAS scores obtained by the control and ketorolac groups, while pain intensity was lower in the dexmedetomidine group. Yu et al. reported the postoperative preventive analgesic effects of dexmedetomidine (29), although they had compared dexmedetomidine and remifentanil infusion in patients undergoing spinal and vertebral surgeries. They suggested that decreased affective-emotional experience might be the cause of reduced pain 48 hours postoperatively. They concluded that dexmedetomidine has preventive analgesia effect on post-operative pain. These findings were also supported in another study (30).

Different factors, which may affect the nociceptive stimuli, such as type as well as length of surgery, level of neuronal damage, type of anesthesia, PCA agent, and post-operative pain control protocols, also influence the findings of studies on various aspects of clinical pain (31, 32).

The extend of tissue trauma in our study was mild to moderate, compared to the above-mentioned studies. However, we obtained favorable results in the dexmedetomidine group, which suggest that the study protocol affects the primary outcomes more than other variables. In line with this finding, a study by Fu et al. (33) showed that the VAS scores and outcomes were more favorable in the dexmedetomidine group.

Different mechanisms for the analgesic effects of dexmedetomidine have been stated, such as prevention of pain impulse transmission through activation of alpha-2 adrenergic receptors at the spinal level (as well as supraspinal and peripheral loci), suppression of the affective-motivational and stress factors of pain, reducing opioid-induced hyperalgesia (34), reduced activation of the sympathetic system (and subsequent attenuation of cytokines and chemokines released due to tissue damage) or inhibiting nuclear factor kappa-β activation (35), which are comparable to the peripheral action of ketorolac. Ketorolac has both central and peripheral activities, which make it a suitable analgesic agent for postoperative pain if administered intraoperatively; the efficacy is lower if administered postoperatively (36). Although the opioid receptor activity of ketorolac has been confirmed (37), the antinociceptive activity of dexmedetomidine was superior in our study due to its longer analgesic effects within 24 hours after surgery.

Based on the findings, the total dose of fentanyl PCA was significantly lower in the dexmedetomidine group than the control group. Similar findings have been announced regarding the opioid-sparing effects in bariatric surgeries (38). In a large study on gastrointestinal surgeries Dexmedetomidine showed a significant opioid sparing effect (39).

In a review study conducted by Jessen Lundorf et al. (40), after screening 2137 records, a meta-analysis was conducted on seven studies, and the overall opioid-sparing effect was reported almost 25% or higher in majority of studies within 24 hours after operation, with no significant difference in postoperative pain. Furthermore, in a recent paper by Tseng et al., the postoperative need for fentanyl PCA was significantly less in the dexmedetomidine group than the control group 24 hours postoperatively (41). Brant et al. had stated that ketorolac had the highest opioid sparing effect among other analgesics (42). In our study, the opioid-sparing effects were almost 30% and 50% in ketorolac and dexmedetomidine groups respectively, compared to the control group.

The pharmacodynamics of dexmedetomidine alone on cardiovascular system is so that the blood pressure accentuates first due to its high plasma concentration and gradually decreases as its plasma concentration attenuates (43). We only had a meaningful decrease of MAP in the early part of the surgery in the dexmedetomidine group. The blood pressures rose gradually towards the end of operation. This is unlike the classic biphasic response seen after a bolus infusion with an initial increase and later decrease of blood pressure due to the sympathetic blockade of dexmedetomidine. Similar results by Seangrung et al which showed an initial decrease in SBP and MAP and heart rate 4 to 10 minutes after intubation. Which was due to the synergistic effects of dexmedetomidine and anesthetic agents used for induction (44).

 It has been stated that dexmedetomidine can cause either decreases or increases in the body temperature, even fever in some cases have been reported too. The range of temperatures have been reported in the range of 35 to 37.8°C (45). In our study the temperatures in the dexmedetomidine group were higher compered to ketorolac and control group. Although different mechanisms such as drug effects on thermoregulation, drug administration-related reactions, pharmacologic drug actions, idiosyncratic responses, and hypersensitivity/ immunologic reactions have been proposed but the exact the explanation for such finding is pending to more studies (46).

It has been reported that the intraoperative infusion of dexmedetomidine can attenuate the occurrence of postoperative nausea and vomiting during recovery due to the reduced amount of opioid consumption perioperatively (47). However, in a study in bariatric surgery patients dexmedetomidine was not associated with decreased incidence of PONV compared to the standard control group (48). Similarly, in our study, we did not find a significant decrease in PONV in D group compared to other groups, and despite meperidine administration for three patients in the recovery period, nausea and vomiting outcomes were almost the same. Similar results were shown by Xu et al. (49).

According to our literature search, no study has yet examined the preventive/preemptive effects of dexmedetomidine and ketorolac comparatively. Although in our study, hemodynamic fluctuations were not prominent, these changes might be related to the side effects of dexmedetomidine, involving α-2A and α-2B receptor subtypes associated with the mode of drug administration (50) Further perspective studies, including new α-2 adrenergic receptor agonists without any hemodynamic fluctuations, can help determine the proper time for administration of different dexmedetomidine doses in order to make better comparisons between different agents and dexmedetomidine regarding the preventive analgesic efficacy.

### 5.1. Limitations

This study has some limitations. First, exact quantification of postoperative bleeding was not performed, as we did not observe any surgical complications, such hematoma, especially in the ketorolac group. Second, it has been methodologically suggested to avoid ketorolac before surgical incisions, since it can inhibit platelet aggregation (51). However, considering the preemptive/preventive analgesia concept and standardization of the study protocol, we applied the drug before induction. Third, we did not measure the total dose of propofol and did not use a bispectral index during operations. Finally, we did not evaluate the patients for more than 24 hours postoperatively, as they were discharged from the hospital within this period. Overall, further follow-up studies can give us a better insight into different modalities to manage acute as well as chronic postoperative pain and the long-term outcomes.

### 5.2. Conclusions

Dexmedetomidine was more effective to decrease pain post-operatively and had a greater opioid sparing effect compared to ketorolac. Post-operatively other outcomes such nausea/vomiting and shivering was comparable.

## Data Availability

The dataset presented in the study is available on request from the corresponding author during submission or after publication.

## Clinical Trial Registration Code:

IRCT20140615018091N9.
